# etymologia:*** Bartonella henselae ***

**DOI:** 10.3201/eid1406.080980

**Published:** 2008-06

[bär′′ tə-nel′ə henz′ ə-lā]

*Bartonella* is a genus of gram-negative bacteria named after Peruvian scientist Alberto Leonardo Barton. He identified a unique bacterium in 1905 during an outbreak among workers building a railway between Lima and La Oroya, a mining town in the Andes. The illness, usually fatal, was characterized by fever and severe anemia. Many of the sick were brought to Guadalupe Hospital in Lima, where Dr. Barton isolated the etiologic agent (which had been transmitted by sandflies) in patients’ blood cells. It was later called *Bartonella bacilliformis*.

The species *B. henselae* was named after Diane Hensel, a technologist in the clinical microbiology laboratory, University Hospitals, Oklahoma City, who in 1985 observed a *Campylobacter*-like organism in blood cultures of HIV-infected patients. The organism was first named *Rochalimaea henselae* and then *B. henselae, *when sequencing showed identity with that genus.

